# Suprasellar chordoid glioma: a report of two cases

**DOI:** 10.20945/2359-3997000000092

**Published:** 2018-10-01

**Authors:** Karina Danilowicz, Santiago Gonzalez Abbati, Soledad Sosa, Florencia Lustig Witis, Gustavo Sevlever

**Affiliations:** 1 Universidad de Buenos Aires Universidad de Buenos Aires Hospital de Clínicas Buenos Aires Argentina Hospital de Clínicas, Universidad de Buenos Aires, Endocrinology, Buenos Aires, Argentina; 2 FLENI Instituto de Investigaciones Neurológicas “Dr. Raúl Carrea” Buenos Aires Argentina Instituto de Investigaciones Neurológicas “Dr. Raúl Carrea”, FLENI, Pathology, Buenos Aires, Argentina

## Abstract

Chordoid glioma (CG) is considered a slow growing glial neoplasm. We report two new cases with endocrinological presentation, management and outcome. Case reports: 1) An 18 year-old female patient was admitted due to headaches, nausea and vomiting and visual abnormalities. She was in amenorrhea. A brain magnetic resonance imaging (MRI) demonstrated a 35 mm-diameter sellar and suprasellar mass. An emergency ventricular peritoneal valve was placed due to obstructive hydrocephalus. Transcraneal surgery was performed. The patient developed central hypothyroidism, adrenal insufficiency and transient diabetes insipidus; she never recovered spontaneous menstrual cycles. Histopathologic study showed cells in cords, inside a mucinous stroma, positive for glial fibrillary acidic protein (GFAP). Due to residual tumor gamma knife radiosurgery was performed. Three years after surgery, the patient is lucid, with hypopituitarism under replacement. 2) A 46 year-old woman complained about a three year-history of amenorrhea, galactorrhea and headache. An MRI showed a solid-cystic sellar mass 40 mm-diameter that extended to the suprasellar cistern. She had hypogonatropic hypogonadism and mild hyperprolactinemia. The tumor mass was removed via nasal endoscopic approach. Histopathological study reported cellular proliferation of glial lineage positive for GFAP. The patient evolved with central hypothyroidism and diabetes insipidus. She was re-operated for fistula and again under the diagnosis of extradural abscess. She evolved with cardiorespiratory descompensation and death, suspected to be due to a thromboembolism. In conclusion, the first case confirms that best treatment for CG is surgery considering radiotherapy as an adjuvant therapy. The other case, on the contrary, illustrates the potentially fatal evolution due to surgical complications.

## INTRODUCTION

Chordoid glioma (CG) is a primary tumor of the central nervous system (1). It was described by Brat et al. in 1998 (2) in a series of 8 cases. In 2007 CG has been included in the World Health Organization (WHO) classification (3) as a neuroepithelial tumor. The incidence is not clear: to our knowledge, there are 99 described cases at the moment.

The name CG is due to the histological similarity to chordoma and the immunostaining with glial fibrillary acidic protein (1). It is a rare neoplasm with a chordoid appearance (4), attached to the hypothalamic or suprasellar area.

We report two new cases with endocrinological presentation, management and outcome, with a review of the literature.

## CASE REPORTS

### Case 1

An 18 year-old female patient was admitted due to a two-month history of intense headaches, nausea and vomiting. She referred visual abnormalities and progressive sleepiness. She was in amenorrhea for a period of 8 months. She did not refer polyuria.

**Figure 1:**
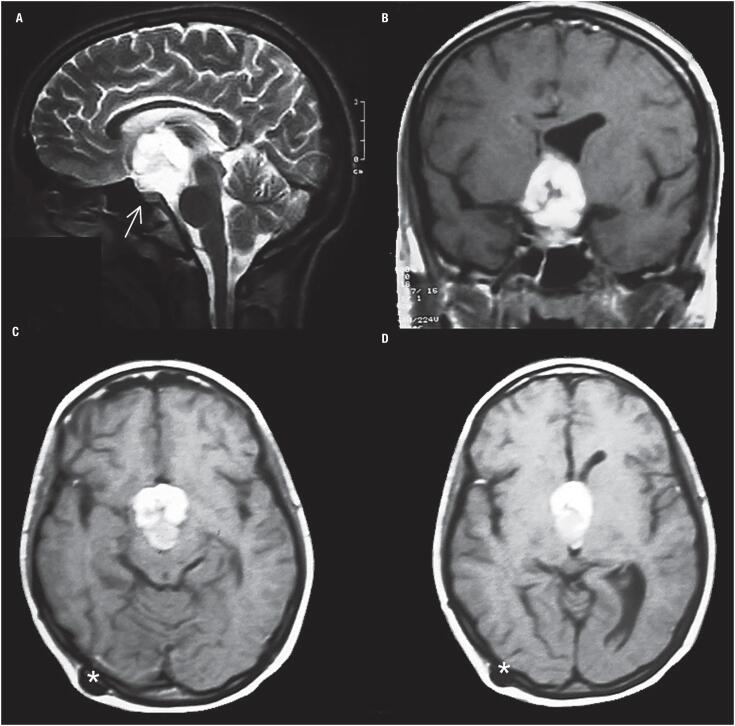
Patient 1. Magnetic resonance imaging findings: sagittal, coronal and axial images. A suprasellar ovoid mass with homogeneous enhancement after gadolinium administration is shown. (A) The sagittal T2 weighted image shows a suprasellar hyperintense ovoid mass with third ventricle extension. Note the normal pituitary gland in a normal sella (arrow). (B) The coronal T1 weighted image shows avid and homogeneous enhancement after gadolinium administration and ventricular asymmetry despite the septostomy and shunt. (C and D) The axial T1 weighted images with gadolinium show the involvement of the tumor in the interpeduncular cistern and the third ventricle. No hydrocephalus was detected due to the ventriculo-peritoneal shunt (asterisks).

A brain magnetic resonance imaging (MRI) evidenced a sellar and suprasellar expansive mass of 35 mm diameter (Figure 1), with intense homogeneous enhancement on T1 weighted imaging after gadolinium administration and hyperintense on T2 weighted imaging. The mass was hyperdense on computed tomography.

Once arrived in our hospital her consciousness worsened and she presented seizures. A ventriculoperitoneal shunt plus endoscopic septostomy with biopsy of the third ventricle mass was done at the same procedure, due to obstructive hydrocephalus. The biopsy initially suggested the diagnosis of chordoid glioma versus pilomixoid astrocytoma. Transcranial surgery was performed by a right pterional approach and trans-lamina terminalis partial resection of the tumor was done. Seven days post-surgery she presented left lung atelectasis requiring respiratory assistance. A pulmonary embolism was ruled out. Postoperatively the patient developed central hypothyroidism, adrenal insufficiency and transitory diabetes insipidus (DI), and she never recovered spontaneous menstrual cycles.

**Figure 2:**
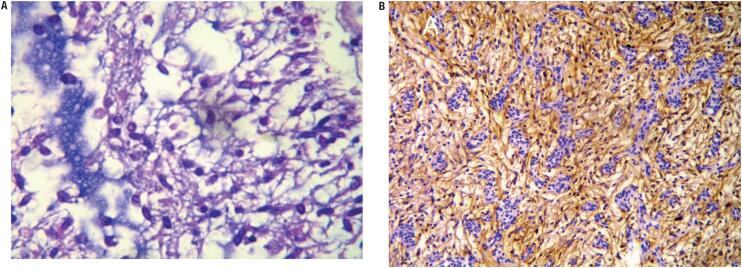
Immunohistochemical findings. (A) Hematoxylin and eosin stain (400x): small cells with circular and oval nucleus, with eosinophilic cytoplasm in a mucinous stroma. (B) Glial fibrillary acidic protein stain (100x): intense and diffuse reactivity for GFAP, in clusters and cords.

Histopathology showed cells with oval nuclei, in cords, inside a mucinous stroma, positive for glial fibrillary acidic protein (GFAP), and negative for neurofilaments and epithelial membrane antigen (EMA). The diagnosis of chordoid glioma was settled (Figure 2).

**Figure 3:**
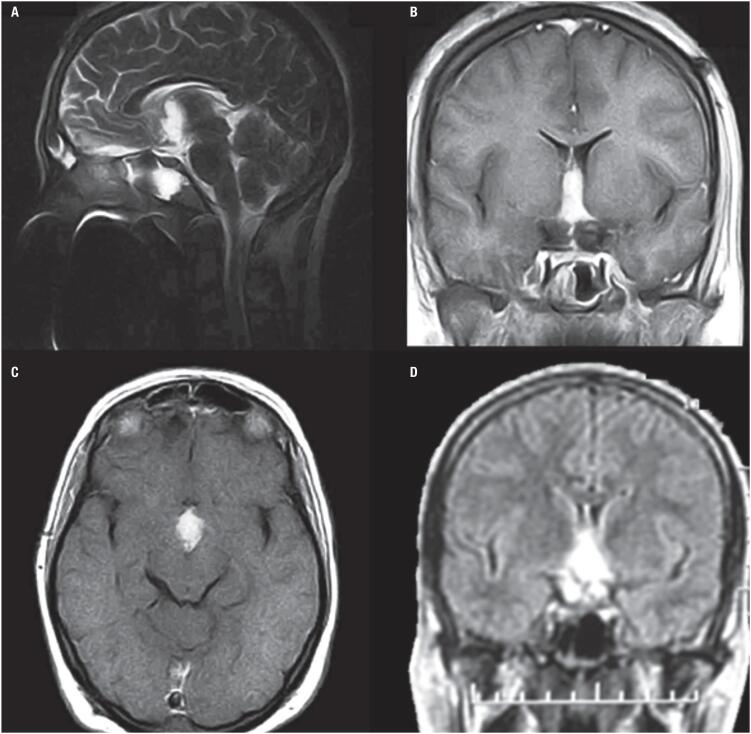
Patient 1. Postoperative and pre-radiotherapy magnetic resonance imaging findings. (A) The sagittal T2 weighted image showed remaining tumor at the third ventricle but without no significant mass effect. (B) The coronal T1 gadolinium weighted image showed the tumor inside the third ventricle but with ventricular symmetry. (C) The axial T1 gadolinium weighted image showed remnant tumor in the interpeduncular cistern. (D) The FLAIR sequence showed spontaneous brightness of the chordoid glioma.

Three months post-surgery she developed poliphagia and memory disturbances. Eighteen months post-surgery, due to residual tumor of 10 per 19 mm (Figure 3), gamma knife radiosurgery was performed.

Three years after surgery, the patient is lucid, with a normal appetite, under replacement therapy with levothyroxine 125 µg/day, hydrocortisone 15 mg/day and etinilestradiol with levonorgestrel.

### Case 2

A 46 year-old woman complained about a three year-history of amenorrhea and galactorrhea. Headache began one year before consultation, but worsened on the last month.

**Figure 4:**
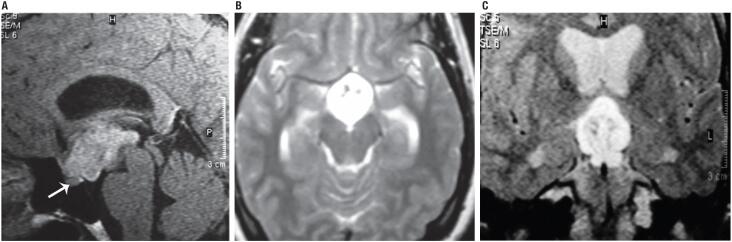
Patient 2. Magnetic resonance imaging findings: sagittal and coronal images. A suprasellar mass with heterogeneous enhancement after gadolinium administration is shown, with hypothalamic involvement and hydrocephalus. (A) The sagittal T1 weighted image shows a suprasellar tumor with gadolinium enhancement compromising the interpeduncular cistern and the third ventricle. The normal pituitary gland is in a normal position (arrow). (B) The axial T2 weighted image shows spontaneous hyperintensity. (C) The coronal FLAIR shows tumor brightness and associated obstructive hydrocephalus.

An MRI showed a solid-cystic sellar mass of 22 × 25 × 40 mm, that extended to the suprasellar cistern, interpeduncular and prepontine region. It was homogeneous with gadolinium enhancement on T1 weighted images. The FLAIR and the T2 weighted sequences showed hyperintensity of the lesion. The normal pituitary gland was in situ below the tumor. Mild supratentorial ventricular dilatation was detected (Figure 4). Visual field examination revealed peripheral temporal scotomies in both eyes.

She had laboratory abnormalities that evidenced hypogonadotropic hypogonadism and hyperprolactinemia without alterations of thyroid and adrenal axis.

**Figure 5:**
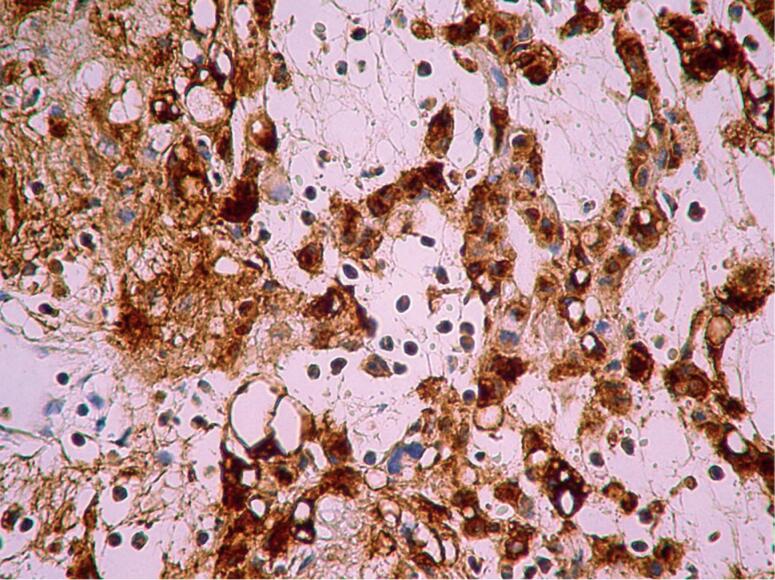
Immunohistochemical findings. Positive cytoplasmic immunostaining for glial fibrillary acidic protein (400 X).

With a presumptive diagnosis of papillary craniopharyngioma, the patient was referred for surgical treatment. The tumor mass was in a retrochiasmatic and third ventricle topography, and was removed by extended endonasal endoscopic approach: an extended transplanum transtubercullum sellae approach was done and a total tumor resection could be achieved. Histopathological study reported cellular proliferation of glial lineage with slight nuclear pleomorphism and moderate diffuse eosinophilic cytoplasm. There was sparse myxoid material and mild lymphoplasmocytic infiltrate. Immunostaining was positive for GFAP, with focal expression of EMA in plasma cells. Proliferation index Ki67 was below 1%. Chordoid glioma was diagnosed (Figure 5).

The patient evolved postoperatively with temporal and spatial disorientation, central hypothyroidism and diabetes insipidus. Treatment with levothyroxine and desmopressin was initiated. She developed cerebrospinal fluid fistula and pioventriculitis. Klebsiella pneumoniae was isolated from cerebrospinal fluid. She was re-operated for fistula closure and a temporary external ventricular shunt was placed. Systemic and intrathecal antibiotic treatment was completed with good response.

Twenty-eight days after the second intervention, the patient presented fever again and an isointense mass in T1 sequence was observed on MRI, near the place where the fistula was repaired. Under the presumptive diagnosis of extradural abscess, she was submitted for rhinosinusal exploration without microbiological isolation. Sixteen days later, she evolved with cardiorespiratory descompensation, suspected to be due to a thromboembolism, and died.

## DISCUSSION

The chordoid glioma is a rare low-grade neoplasm mostly located in the third ventricle. Obstructive hydrocephalus may develop due to its location (5). The CG is considered a glial neoplasm (6). CG is more commonly found in adult women, rarely in the pediatric age group (7-9). Our first case is more unusual since CG occurred in an 18 year-old woman.

The CG mostly presents insidiously (74%) (10), rarely acutely (5). As described by Desouza and cols. (10) the CG mostly presents with headaches (40%) and visual defects (30%). Other symptoms described in these patients are memory deficits (24%), ataxia or incoordination (10%), endocrine disturbances with gonadotropic alterations as the most frequent (10%) (10). Most of the cases described are multi-symptomatic (78%), being the most typical one, the presence of visual defects (10). In few patients, CG has been detected incidentally (3.7%) (1). In 2002 Oda and cols. (11) describe a case of a male with a two-year history of voracious appetite and progressive memory impairment three months prior to evaluation. Both of our cases presented severe headaches, with hypogonadism as the endocrine abnormality, and obstructive hydrocephalus in coincidence with the most common findings reported.

CG should be included in the differential diagnosis of third ventricle's masses. The most characteristic image suggestive of CG on MRI is an ovoid well-defined mass on the anterior region of the third ventricle or sellar region extending towards the hypothalamus. CG is usually isointense in T1 weighted images, with strong uniform contrast enhancement and with bilateral vasogenic edema (10). They may have cystic components (25%) and, though unusual, calcifications and hemorrhage can be found (10). On CT scans CG is hyperdense with homogeneous contrast enhancement. The lesion in our cases was typically oval, located in the suprasellar area, with a strong contrast enhancement on MRI, and hyperdense on CT in case 1. In case 1, the mass enhanced homogeneously after gadolinium administration and was hyperintense on T2 weighted image, with third ventricle extension. Postoperatively, on T1 gadolinium weighted image the remnant tumor was inside the third ventricle with ventricular symmetry. In case 2, the suprasellar mass presented with heterogeneous enhancement after gadolinium administration with hypothalamic involvement and hydrocephalus on T1 weighted image, hyperintense on T2 weighted image. In the literature CG has been usually described as well circumscribed, ovoid in shape, isointense on T1 weighted MRI scans, with intense and uniform enhancement (12,13). Liu and cols. (14) found 63% of CG isointense on T1 weighted images, 70% with uniform enhancement and 42% hyperintense on T2 weighted images, aiding these findings in the differential diagnosis.

The most difficult differential diagnosis is with craniopharyngiomas which have solid parts in adults. This was our presumptive diagnosis in case 2, inducing a transphenoidal approach.

Tumors that originate in the anterior portion of the third ventricle should be considered. Pilocytic astrocytomas grow along optic structures and affect younger individuals, as it occurs in germinomas. Suprasellar meningiomas have the typical dural prolongation. Ependymomas have heterogeneous enhancement, frequent calcifications and internal hemorrhage (15).

Histopathological findings consist of clusters and cords of epithelioid cells with abundant eosinophilic cytoplasm in a mucinous stroma with infiltrates of mature lymphocytes and plasma cells (2). By immunohistochemistry, they are positive for GFAP indicating the tumor's glial origin (16). CG was confirmed in our cases due to the microscopic findings as well as the immunophenotype. TTF-1 transcription factor has been demonstrated to be constantly expressed in a series of 17 cases of CG of the third ventricle (17). Though not specific, this marker could help in diagnosis of this rare tumor.

The treatment of choice is transcranial surgery, but incomplete resections are not uncommon. Mean time from onset of symptoms to surgical management has been described in 22.1 months (range 0-20 years) (18). In a review analyzing 54 operated patients, 22.7% recurred after partial resection and 74% died over a mean follow-up of 10.2 months (19). In a recent systematic review of 81 patients, the extent of surgical resection was reported in 75, of which 10.7% received biopsy, 45.3% subtotal resection and 44% gross total resection (18). Ventricular valves are often needed due to obstructive hydrocephalus as in our two cases.

Surgical approach has not been specified in many cases. In our first case the transcranial approach was chosen based on histopathologic possibilities obtained through the biopsy, since the best option for both diagnosis settled is the surgical resection through the transcranial approach. Our second case was approached endoscopally since was initially interpreted as a craniopharyngioma. There are no reports about the indication of extended endoscopic endonasal approach in gliomas, being our case the first described using this surgical approach. Currently, the most direct route to the suprasellar region is through an extended approach over the sella: the transplanum-transtuberculum approach, which allows the dissection of midline tumors over the pituitary gland and below the optic chiasm, without the need for brain retraction. This approach allows to follow the tumor mass located within the third ventricle and a visual control from below of the entire third ventricle, which cannot be obtained by any transcranial approach. Thus, the endoscopic approach has been used with more frequency in recent years for the treatment of tumors, especially craniopharingyomas, extending from the suprasellar region into the third ventricle (20).

Surgical complications of endoscopic endonasal resection of tumors that extend into the third ventricle include neurological deficits as a result of direct neural tissue trauma or vascular compromise, pituitary hormonal dysfunction as a result of injury to the pituitary stalk or hypothalamus, and cognitive and psychological abnormalities as a result of injury to the frontal/temporal lobes or hypothalamus. Cerebrospinal fluid leakage and sinonasal morbidity are the most common approach-specific complications (20). The most common postoperative complication described in the surgical treatment of CG is hypothalamic dysfunction, mostly diabetes insipidus and less frequently syndrome of inappropriate antidiuretic hormone (18). Short-term memory deficits are present in 8.8% (18). The trans-lamina terminalis is associated with less postoperative morbidity, but without statistically significance (18). Post-operative complications in CG are DI (31%), amnesia (26%) and pulmonary embolism (15%) as the most frequent (10), with 38.3% of complications (18). Mortality in the immediate postoperative period is described in 32%, mainly due to thromboembolism, being higher after gross total resection. Ampie and cols. (18) describe in a comprehensive review of 81 CG, 18 deaths (22.2%) at last follow up, mostly not related to disease progression, half of them within 18 days of surgery. Non-fatal postoperative complications are hypothalamic disorders and mental alterations (19). Gan and cols. (21) have recently described a high prevalence of obesity in a series of pediatric low grade gliomas affecting the optic pathway, hypothalamus and suprasellar areas (50% at 20 y). However, no statistical significant difference in the rate of overall complications has been shown between biopsy/ partial or gross resection (p = 0.82) (18). However, the most robust predictor of tumor control was gross total resection (18). In our first case surgery was performed with a partial resection, with few events on follow-up. In the immediate postoperative period, endocrine dysfunction was confirmed. Radiosurgery was indicated due to the remnant. Three years post treatment, the patient has only endocrine sequelae and no regrowth of the CG. The second case developed postoperatively infectious complications and finally died due to the presumptive diagnosis of thromboembolism. The high morbidity rate described in the surgical management of CG could be the reason for this fatal evolution, though more experience is needed to conclude about the benefits and risks of the endoscopic endonasal approach.

Radiotherapy may be indicated when the resection is not complete. Progression or recurrence occurs in 30% of the patients; in these cases prognosis is usually bad. Nakajima and cols. (22) presented a case where the CG was resected and later treated by stereotactic radiosurgery. No regrowth was observed at 2-year follow-up. Kobayashi and cols. (19) described three cases of CG of the third ventricle with special emphasis on the effects of planned microsurgery and low dose stereotactic radiosurgery. However, other authors showed recurrences after radiotherapy (23,24). Our first case is another one where gamma knife has been used; 3 year-follow-up shows no recurrence with a stable remnant. However, the real efficacy of radiotherapy is still unclear.

In these cases surgery was the main treatment by a transcranial or endonasal endoscopic approach, considering radiosurgery as an adjuvant therapy particularly in those cases of partial resections. Hypopituitarism is a plausible adverse event. However, CG might fatally evolve due to surgical complications. This postoperative mortality in the management of a low-grade tumor should be taken into consideration in therapeutic decisions. Since the goal of treatment is complete total resection pursuing a curative intention, but due to the high perioperative morbidity, options should be balanced to define an individualized therapeutic approach. More experience is needed to bring conclusions about the endoscopic endonasal approach for the management of CG.
